# Maternal nutritional status and the risk of metabolic syndrome in pregnancy: implications for public health nutrition

**DOI:** 10.3389/fpubh.2026.1862412

**Published:** 2026-07-01

**Authors:** Yu Wu, Zhengxiang Gao, Yu Gou, Lingyi Yan, Yifei Duan

**Affiliations:** 1 Department of Laboratory Medicine, West China Second University Hospital, Sichuan University, Chengdu, Sichuan, China; 2 Key Laboratory of Birth Defects and Related Diseases of Women and Children, Sichuan University, Ministry of Education, Chengdu, Sichuan, China

**Keywords:** BMI, dietary intake, fibre intake, maternal nutrition, metabolic syndrome, pregnancy

## Abstract

**Background:**

Maternal nutritional status is considered essential for metabolic adaptation during pregnancy. Both malnutrition and obesity are associated with metabolic abnormalities; however, the link between these factors and metabolic syndrome (MetS) in pregnancy remains poorly studied from a public health perspective.

**Aim:**

The aim of the current study was to evaluate the association between nutritional status and the risk of metabolic syndrome in pregnant females.

**Methods:**

In a retrospective observational study, data were collected from the medical records of 850 pregnant women from tertiary care hospitals between November 2023 and May 2025. Assessment of nutritional status was carried out using dietary and body mass index (BMI). MetS was diagnosed based on the presence of three or more metabolic conditions. Logistic regression was employed to identify significant risk factors, adjusting for various covariates.

**Results:**

MetS was diagnosed in 24.7% of cases. Patients with MetS were found to have higher BMI, dietary calorie intake, fat, and sugar consumption, and lower fibre consumption (*p* < 0.001). Risk factors of MetS included poor nutritional habits (AOR = 2.45), insufficient fibre intake (AOR = 2.12), increased BMI (AOR = 2.76), and excessive dietary fat intake (AOR = 1.89). There appeared to be a dose–response effect between fibre intake and MetS.

**Conclusion:**

Nutrition status is significantly associated with the risk of MetS during pregnancy. Findings suggest that nutritional evaluation and intervention are important strategies in public health nutrition.

## 1 Introduction

Nutritional conditions during pregnancy are a key determinant of maternal and offspring health, especially with respect to metabolic syndrome (MetS). Metabolic syndrome, characterised by a series of interacting metabolic disorders such as insulin resistance, central obesity, dyslipidemia and hypertension, has been more common in women of reproductive age. Pregnancy is a metabolically dynamic condition, and an inappropriate nutritional status may contribute to metabolic alterations, making the person susceptible to metabolic disorders and poor pregnancy outcomes (1, 2). In addition, women with pregnancy complications have a higher risk of developing metabolic syndrome in the future, which underscores the importance of pregnancy as an important period to measure metabolic health (3).

One of the key modifiable factors that affects the metabolic health of a pregnant woman is maternal diet. There is evidence that high energy intake and imbalances in protein, fat, and carbohydrate ratios contribute to insulin resistance, dyslipidemia, and increased metabolic risk (4). Conversely, healthy eating habits, especially Mediterranean diets, have been associated with a lower incidence of metabolic syndrome and improved glycemic control during the postpartum phase (5). Also, a higher intake of dietary fibres has been shown to positively impact maternal metabolic parameters, including insulin sensitivity and systemic inflammation (6).

Notably, the quality of periconceptional diets has also been associated with the probability of developing metabolic syndrome many years later during pregnancy, making it crucial to consider early nutritional exposures (7). In addition to macronutrient content, the nutritional status of the maternal diet is also affected by dietary diversity, micronutrient adequacy, and supplementation. There has been a correlation between limited dietary diversity during pregnancy and negative birth outcomes and metabolic vulnerability (8). The imbalance of micronutrients, especially deficiencies of essential trace elements, may lead to dysfunctional metabolic pathways and the development of metabolic syndrome in children (9).

Moreover, evidence from low- and middle-income countries emphasizes that adequate maternal nutrition and supplementation are essential for achieving improved fetal and neonatal outcomes, which are closely related to long-term metabolic outcomes (10). Beyond micronutrient adequacy and dietary diversity, broader metabolic disturbances associated with maternal overnutrition have also emerged as major contributors to adverse pregnancy outcomes. Some of the significant risk factors leading to metabolic syndrome in and after pregnancy are maternal obesity and gestational diabetes mellitus (GDM). The obese condition triggers a chronic inflammatory and insulin-resistant condition known as a so-called metabolic storm that increases metabolic disruptions in pregnancy (2). Women who have had GDM before are especially vulnerable to developing metabolic syndrome, and maternal dietary issues are also contributing factors (11). Metabolic syndrome has also been very prevalent among women who have GDM, especially in the second trimester of pregnancy (12). Moreover, metabolic profiling in early pregnancy has shown unique metabolic signatures of obesity that can predict the development of later complications and metabolic consequences (13).

Other exposures during fetal development can cause long-term alterations in gene expression, endocrine activity, and metabolism, thereby affecting susceptibility to disease in late adulthood. The mediation of these processes is possible through epigenetic, placental, and nutrient-supply changes in the intrauterine environment (14). Moreover, there are also intergenerational consequences of maternal nutrition. There is a close relationship between maternal dietary trends and metabolic health, as well as the risk of non-communicable disease development in children, such as obesity, insulin resistance, and metabolic syndrome (15). Poor quality of the diet and unhealthy eating habits, including night eating, are the lifestyle factors associated with negative metabolic outcomes in children, which further support the long-term effects of maternal nutritional exposures (16).

Although research on maternal nutrition and metabolic health has increased, several crucial gaps remain in understanding how overall nutritional status affects the development of metabolic syndrome during pregnancy. Much of the current literature focuses on individual nutrients or specific components of the diet and does not consider maternal nutrition as a whole, integrated approach. Moreover, variations in study design, population characteristics, and timing of nutritional evaluation complicate the generalisation of conclusions. This also lacks context-specific evidence, especially from low- and middle-income contexts, where dietary habits and access to healthcare vary greatly. Thus, this research aims to assess the relationship between maternal nutritional status and the incidence of maternal metabolic syndrome during pregnancy, to identify key nutritional determinants that may contribute to metabolic risk, and to facilitate the development of specific nutritional interventions to reduce maternal and infant morbidity.

## 2 Methodology

### 2.1 Study design and setting

This retrospective observational study aimed to examine the relationship between maternal nutritional status and the occurrence of metabolic syndrome during pregnancy. Medical records of tertiary care hospitals and related antenatal clinics were used in the study. Electronic health records and archived patient files from a specific study period (between November 2023 and May 2025) were accessed as data sources. The retrospective design enabled a universal assessment of systematically collected clinical, biochemical, and dietary data, reflecting the actual state of maternal health.

### 2.2 Study population and the sampling strategy

**Figure 1:**
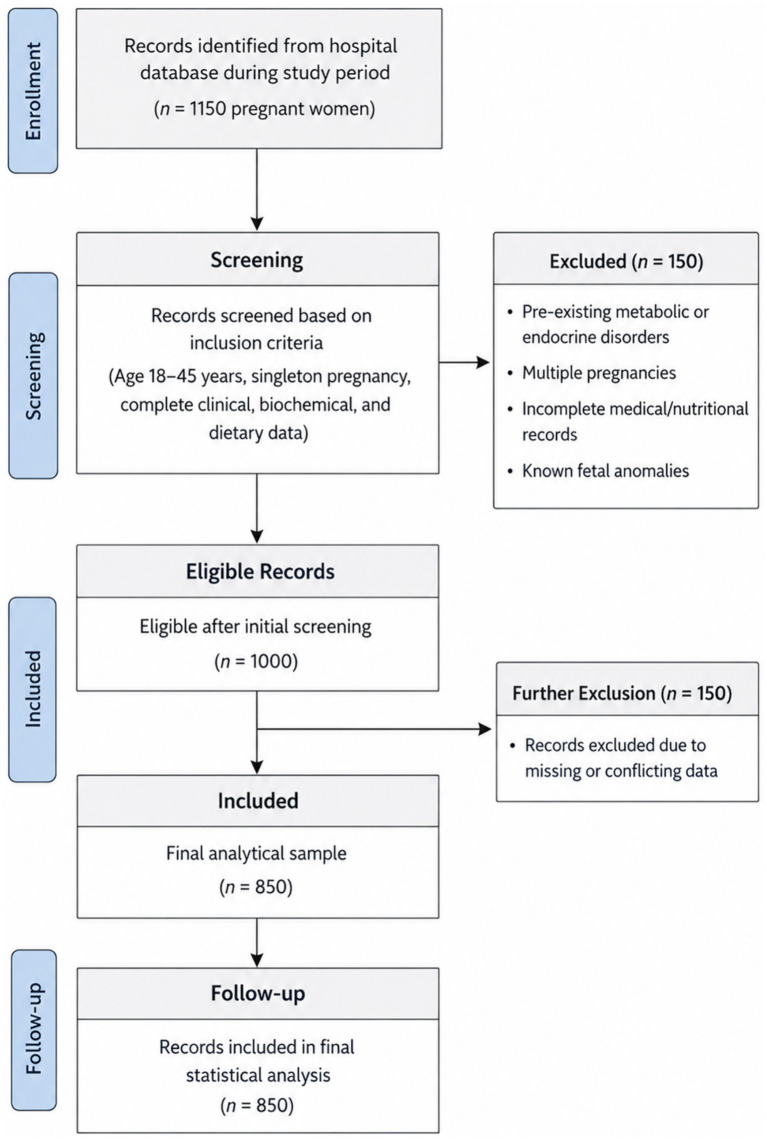
Participant selection flow diagram.

After systematic retrospective screening, 850 pregnant women were incorporated in the final analysis. First, about 1,150 antenatal records were identified in hospital databases during the study period. These documents were screened on pre-determined inclusion and exclusion criteria, as shown in Figure 1.

Women aged 18 to 45 years with singleton pregnancies and complete clinical, biochemical, and dietary records were considered eligible. The exclusion criteria included pre-existing metabolic or endocrine disorders, multiple pregnancies, incomplete medical or nutritional records, and pregnancies complicated by known fetal anomalies.

Following preliminary screening, about 1,000 records were eligible. Subsequently, 150 records were excluded because of missing or conflicting data, resulting in a final analytical sample of 850 participants. The participant selection process followed a structured sequence of identification, eligibility screening, exclusion, and final inclusion to ensure methodological transparency and minimize selection bias.

### 2.3 Sample size justification

The sample size of 850 was deemed sufficient to identify statistically significant relationships between maternal nutritional status and metabolic syndrome, with adequate power. Since the study was retrospective, the sample was selected based on the number of eligible records available at the time of the study. The number of individuals sampled was sufficient to conduct the multivariate regression analysis, which holds the estimates constant and provides greater generalizability of the results.

### 2.4 Data collection procedures

A standardized data abstraction protocol was used to extract data to ensure consistency and accuracy. Medical records provided information on maternal demographics, obstetric history, anthropometric data, biochemical data, and dietary intake.

Routine antenatal assessments provided anthropometric and clinical measurements, such as body weight, height, and blood pressure. Laboratory reports gave data on biochemical values (such as fasting blood glucose, lipid profile (triglycerides and HDL cholesterol), and other important values). The dietary intake data were obtained during the standard nutritional evaluation work-up of pregnant women at their routine antenatal visits by trained health care workers, using structured dietary assessment forms completed prospectively. Data on average daily calorie intake, macronutrient composition, dietary fibre, and processed food consumption were retrospectively retrieved for the current study from 24-h dietary recall and food-frequency questionnaires, which served as the main dietary assessment methods.

The clinical, biochemical, anthropometric, and dietary parameters analyzed in the current study were collected mostly at the routine second-trimester prenatal check-up, when metabolic screening is commonly performed. Measurements were preferentially taken as close as possible to the time of the metabolic evaluation for participants with multiple antenatal records, to ensure good temporal consistency.

### 2.5 Assessment of maternal nutritional status

Nutritional status was determined using anthropometric measurements and a routine dietary intake assessment was collected as part of antenatal care. Standardized 24 h dietary recall records and food-frequency reporting were used to gather dietary intake information during regular antenatal nutritional evaluations consistent with established maternal nutrition assessment approaches (17). Nutritional intake estimates were derived from routinely documented clinical dietary assessments rather than from a dedicated research-administered validated food frequency questionnaire. The assessments included estimated daily caloric intake, macronutrient consumption patterns, fibre intake, and processed food consumption patterns. Nutritional adequacy was determined based on general maternal nutrition guidelines for pregnancy, including adequate caloric intake balance, macronutrient proportions, and adequate dietary fibre intake, as well as clinical nutritional assessment during pregnancy (18).

Dietary quality was determined in accordance with conventional prenatal nutritional recommendations, which included an adequate daily intake of dietary fibre, a balanced macronutrient distribution, moderate sugar and saturated fat intake, and reduced consumption of processed foods. Individuals who met most of these dietary requirements were classified as having “adequate diet quality,” while those who had several nutritional imbalances, such as low fibre intake and high consumption of processed foods or sugar, were classified as having “inadequate diet quality” or “poor nutritional habits.” Nutritional classification was based on routinely recorded prenatal dietary assessments and established gestational dietary recommendations.

Anthropometric measurements included body mass index (BMI), another measure of nutritional status. This combined approach enabled a comprehensive evaluation of both diet quality and physiological nutritional status. Dietary evaluations were conducted using standardized clinical documentation procedures during routine antenatal visits.

### 2.6 Definition and assessment of metabolic syndrome

The definition of metabolic syndrome in pregnancy was based on modified clinical criteria adapted to the physiology of pregnancy and the internationally recognised definitions of metabolic syndrome (19). At least three of the following were required for diagnosis: elevated fasting glucose (fasting glucose level ≥95 mg/dL), high triglycerides (fasting triglycerides ≥150 mg/dL), low HDL (fasting HDL level <50 mg/dL), and elevated blood pressure (fasting SBP ≥ 130 mmHg or FSBP ≥85 mmHg).

Clinical and biochemical measurements used for metabolic syndrome classification were obtained during routine antenatal assessments, primarily in the second trimester of pregnancy, when metabolic screening is commonly performed in standard prenatal care.

Anthropometric indicators, such as maternal BMI, were evaluated separately as exposure-related nutrition variables but not included in the diagnostic regression model. Access to waist circumference was not reported in all retrospective clinical medical records and was therefore not included in the final operational definition.

The overall dietary balance recorded during the antenatal nutrition assessment was used to classify dietary quality. Dietary intake was relatively well balanced, with adequate calorie intake, moderate fat and sugar intake, sufficient dietary fibre intake, and regular intake of nutrient-diverse foods, indicating adequate diet quality. High consumption of processed foods, sugar, or fat, with low consumption of fibre, was considered an inadequate diet.

To reduce temporal variability between clinical and nutritional variables, metabolic syndrome classification was performed using measurements taken during the same period of the antenatal assessment, where possible.

### 2.7 Key variables and operational definitions

Operationally defined maternal nutritional status was a composite measure of dietary intake adequacy and anthropometric measures, especially BMI. Adequate nutritional status was defined as dietary intake in accordance with recommended guidelines, whereas inadequate status was defined as deficiencies or imbalances in the dietary intake of macro- or micronutrients.

The operational definition of metabolic syndrome was the presence of at least three metabolic abnormalities, such as hyperglycemia, dyslipidemia (elevated triglycerides and/or lowered HDL cholesterol), and elevated blood pressure, according to pregnancy-adapted clinical thresholds. Hypertension, and increased adiposity.

Maternal BMI recorded during antenatal assessment was used as an indicator of maternal nutritional status and analyzed as an exposure-related variable rather than a direct diagnostic component of metabolic syndrome. Blood pressure was measured using systolic and diastolic readings during clinical visits. Dyslipidemia was defined as triglyceride and HDL cholesterol levels above clinically relevant thresholds. Dietary fibre intake was classified as low, moderate and adequate according to the recorded daily intake.

Nutritional habits are operationally defined as excessive consumption of processed foods, refined sugars, and high-fat foods, and low dietary fibre intake.

### 2.8 Control of confounding variables

Confounding variables were identified based on clinical relevance and prior literature examining maternal metabolic risk during pregnancy. Confounders that were included in the analysis were maternal age, parity, education level, residence, family history of diabetes, physical activity status, and maternal BMI. Physical inactivity was defined as the absence of regular moderate physical activity recorded in antenatal assessment records. A binary indicator was created to denote family history of diabetes (Yes/No) based on the presence of documented first degree familial diabetes history. Multivariable logistic regression analyses were performed to estimate the independent association between maternal nutritional status and metabolic syndrome, after accounting for the confounding effects of these covariates.

### 2.9 Handling of missing data

Records were scanned for completeness of the following data before analysis: demographic, biochemical, anthropometric and dietary. Records were assessed for eligibility, and those with significant missing data elements that could affect the exposure and/or the outcome of primary interest were excluded. There were only a few missing values, and these were handled using pairwise deletion, which allowed the maximum number of available observations in each analysis. There was little missing data for each variable, and it would not have significantly altered the composition of the overall sample. Since the missingness rate was relatively low in this study, no multiple imputation procedures were performed.

### 2.10 Statistical analysis

IBM SPSS Statistics version 26.0 (IBM Corp., Armonk, NY, USA) was used to perform statistical analysis. Continuous variables were evaluated and reported as means with standard deviations, medians with interquartile ranges, or frequencies and percentages. Independent-samples *t*-tests or Mann–Whitney *U* tests (continuous variables) and chi-square tests (categorical variables) were used to compare participants with and without metabolic syndrome. Multivariate logistic regression was used to assess the independent association between maternal nutritional status and metabolic syndrome after adjusting for confounding variables. Covariates were added to the adjusted regression model on an *a priori* basis and for biological plausibility. Clinically relevant nutritional thresholds from prenatal nutritional guidelines and previously published maternal nutrition research were used to categorise dietary variables. They were reported as adjusted odds ratios with 95% confidence intervals, and *p*-values below 0.05 were considered statistically significant. Goodness-of-fit and multicollinearity were measured using model diagnostics.

### 2.11 Ethical considerations

This study was approved by the Medical Research Ethics Committee of West China Second University Hospital (Approval Number: 24H0689), and the research was carried out in accordance with the ethical principles stipulated in the Declaration of Helsinki. Since this study was a retrospective analysis of anonymized patient records, there was no need to use informed consent.

## 3 Results

### 3.1 Study population characteristics

Among 850 participants included, about 24.7% (*n* ≈ 210) were diagnosed with metabolic syndrome (MetS), and 75.3% (*n* ≈ 640) did not fit the diagnostic criteria. Table 1 revealed that women with MetS were older (mean 32 vs. 29 years), heavier (approximately 30 vs. approximately 25 kg/m^2^), and had a significantly higher prevalence of metabolic risk factors (including physical inactivity and a positive family history of diabetes). There were also socioeconomic and lifestyle variations, whereby lower education and less physical activity were more prevalent among the MetS group.

**Table 1:** Baseline demographic, socioeconomic, and clinical characteristics (*n* = 850).

Variable	Total	MetS (*n* ≈ 210)	Non-MetS (*n* ≈ 640)	*p*-value
Age (years)	29.8 ± 5.4	32.1 ± 5.0	28.9 ± 5.2	<0.001
BMI (kg/m^2^)	26.7 ± 4.8	30.2 ± 5.1	25.4 ± 4.2	<0.001
Multiparity (%)	58.6	66.2	55.9	0.012
Education (≥secondary %)	54.2	46.8	56.7	0.018
Urban residence (%)	61.3	64.5	60.2	0.210
Family history of diabetes (%)	34.7	48.1	30.2	<0.001
Physical inactivity (%)	41.5	55.3	36.8	<0.001
Smoking exposure (%)	9.6	13.4	8.2	0.031

Values are mean ± SD or percentages. This table shows that both biological factors (BMI, age) and modifiable lifestyle factors (physical inactivity, education level) are strongly related to metabolic syndrome. The fact that these determinants are clustered indicates that there is a multifactorial risk environment of metabolic syndrome during pregnancy.

### 3.2 Nutritional status and dietary intake patterns

There were considerable variations in the dietary consumption between groups, as shown in Table 2. MetS women consumed more calories (≈2,520 kcal vs. ≈ 2,280 kcal) and a higher percentage of calories from fat and refined carbohydrates. Protein consumption and fibre intake were significantly lower among women with MetS (16.2 ± 5.4 g/day vs. 23.4 ± 6.1 g/day). Interestingly, a small proportion of women with MetS met the dietary criteria (28%), compared with more than half of the non-MetS group.

**Table 2:** Dietary intake and nutritional status assessment.

Variable	Total	MetS	Non-MetS	*p*-value
Total energy (kcal/day)	2,350 ± 410	2,520 ± 430	2,280 ± 390	<0.001
Carbohydrates (%)	55.2 ± 6.1	58.4 ± 6.5	54.1 ± 5.8	<0.001
Protein (%)	15.8 ± 3.2	14.2 ± 3.0	16.3 ± 3.1	<0.001
Fat (%)	29.0 ± 5.4	31.6 ± 5.7	28.1 ± 5.0	<0.001
Fibre (g/day)	21.5 ± 6.8	16.2 ± 5.4	23.4 ± 6.1	<0.001
Sugar intake (g/day)	78.6 ± 22.3	92.1 ± 25.4	73.2 ± 19.8	<0.001
Processed food intake (%)	38.4	52.7	33.2	<0.001
Adequate diet quality (%)	46.8	28.1	52.9	<0.001

Table 2 clearly shows that low dietary quality is closely related to metabolic syndrome. The rise in the consumption of fats, sugars, and processed foods, and the decline in fibre intake, indicate a dietary pattern that leads to metabolic dysregulation. Balanced dietary intake patterns reported during antenatal nutrition assessments were used to define adequate diet quality, including proper caloric distribution, reduced intake of processed foods, and adequate fibre intake.

### 3.3 Prevalence and clustering of metabolic syndrome components

The distribution of individual metabolic abnormalities is shown in Table 3. High triglycerides (≈36.9) and high BMI (≈42.6) were the most common abnormalities among all participants. Clustering was also observed in the MetS group, where more than two-thirds of individuals exhibited multiple metabolic derangements concomitantly.

**Table 3:** Prevalence and clustering of metabolic syndrome components.

Component	Total (%)	MetS (%)	Non-MetS (%)	*p*-value
Elevated fasting glucose	28.5	62.3	17.4	<0.001
High triglycerides	36.9	71.5	25.1	<0.001
Low HDL-C	31.4	65.2	20.3	<0.001
Hypertension	33.7	68.9	22.5	<0.001
Elevated BMI	42.6	78.1	31.2	<0.001
≥3 components present (%)	24.7	100	–	–

The clustering effect demonstrates that metabolic syndrome cannot be caused by a single abnormality but by a combination of metabolic disruptions, with lipid abnormalities and obesity being of key importance.

**Figure 2:**
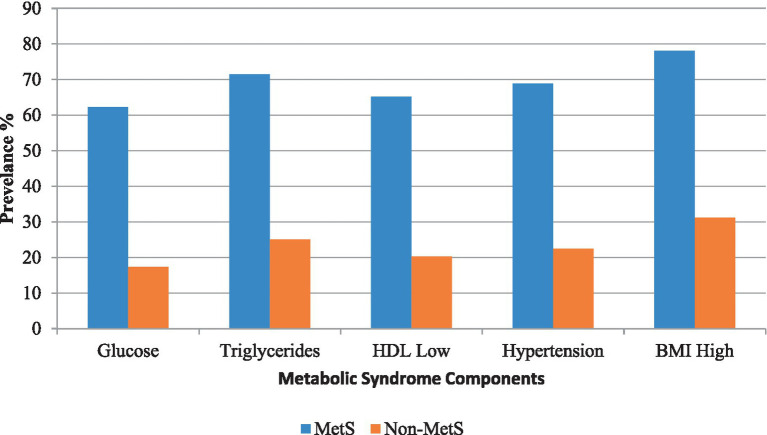
Prevalence of individual metabolic abnormalities among pregnant women with and without metabolic syndrome (MetS).

The percentages of participants with high fasting glucose, high triglycerides (TG), low HDL cholesterol (HDL-C), hypertension, and high BMI are shown in Figure 2. The data are given on women with MetS (blue bars) and non-MetS participants (orange bars). The data show that multiple metabolic abnormalities are more clustered in the MetS group, suggesting that metabolic syndrome is not defined by individual metabolic abnormalities but by their interplay.

### 3.4 Association between maternal nutritional status and metabolic syndrome

Table 4 indicates that multivariate logistic regression analysis revealed that maternal nutritional status was a strong independent predictor of metabolic syndrome even after controlling for confounders. Low diet quality doubled the risk of MetS, and maternal overweight/obesity and low dietary fibre intake remained strong independent predictors of metabolic syndrome after adjustment for confounding variables.

**Table 4:** Multivariate logistic regression model for metabolic syndrome (*n* = 850).

Variable	AOR	95% CI	*p*-value
Inadequate diet quality	2.45	1.78–3.36	<0.001
Low fibre intake	2.12	1.54–2.91	<0.001
High fat intake	1.89	1.34–2.67	<0.001
High sugar intake	1.76	1.28–2.41	<0.001
Maternal overweight/obesity (BMI ≥ 25 kg/m^2^)	2.76	2.01–3.79	<0.001
Physical inactivity	1.68	1.21–2.32	0.002
Family history of diabetes	1.59	1.13–2.23	0.006

Adjusted for maternal age, parity, education level, residence, physical activity status, family history of diabetes, and maternal BMI.

The multivariable regression model demonstrated overall statistical significance (model *χ*^2^ = 168.4, *p* < 0.001) with acceptable explanatory power (Nagelkerke *R*^2^ = 0.38). The Hosmer–Lemeshow goodness-of-fit test indicated adequate model calibration (*χ*^2^ = 6.21, *p* = 0.62). Multicollinearity assessment showed acceptable variance inflation factor values (<2.5 for all variables), indicating no substantial multicollinearity among the predictors.

**Figure 3:**
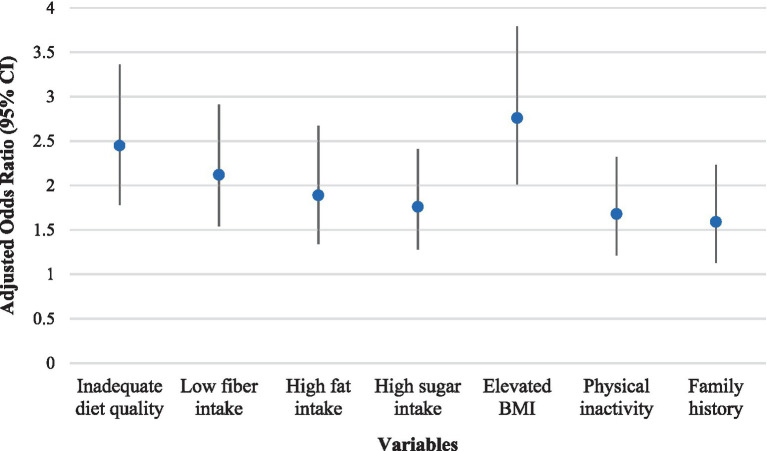
Multivariate logistic regression analysis.

The regression model exhibits high predictive power, explaining a high percentage of the variance in the occurrence of metabolic syndrome. Nutritional factors are independent of the confounder, supporting their clinical significance, as shown in Figure 3. Additional model evaluation demonstrated that the associations between dietary quality, fibre intake, and metabolic syndrome remained directionally consistent after exclusion of adiposity-related variables from exploratory analyses.

Forest plot with adjusted odds ratios (AOR) and 95% confidence intervals of the significant predictors of metabolic syndrome. Poor quality of diet, low level of fibre, excess intake of fat and sugar, high BMI, lack of physical activity and family history of diabetes were found to be independently linked with high odds of metabolic syndrome. The vertical reference line shows an odds ratio of 1.0.

### 3.5 Stratified analysis of dietary fibre and metabolic outcomes

There was a graded inverse correlation between metabolic abnormalities and dietary fibre intake, as shown in Table 5. Women who consumed a sufficient amount of fibre were found to have a much lower prevalence of metabolic syndrome (≈15.9%) as compared to women with low consumption (≈34.8%).

**Table 5:** Fibre intake and associated metabolic risk.

Fibre intake	MetS (%)	High TG (%)	Low HDL (%)	Elevated glucose (%)	Hypertension (%)
Low	34.8	49.2	44.1	38.7	41.5
Moderate	24.6	36.5	30.2	27.9	32.1
Adequate	15.9	22.4	18.7	19.3	21.6

This table illustrates a dose–response relationship, in which the larger the intake of fibre, the larger the metabolic risk reduction. This favours the protective effect of dietary fibre in regulating lipid metabolism and glycemia.

## 4 Discussion

The current research indicates that maternal nutritional status has a strong correlation with the incidence of metabolic syndrome during pregnancy and that dietary and lifestyle changes play a crucial role in determining whether a pregnant woman will develop metabolic syndrome. Poorly-nourished women in our study were more likely to develop metabolic syndrome, which agrees with the results of Habibi et al. (20), who found that unfavourable metabolic profiles in pregnancy are closely associated with gestational metabolic disorders. Moreover, the rising rates of metabolic syndrome in pregnant women, as pointed out by Mohebi et al. (21), further emphasise the growing interest in the topic of maternal metabolic health and its role in pregnancy complications.

The findings of our study are also consistent with those of Li et al. (22), who showed that metabolic syndrome is a significant risk factor for negative pregnancy outcomes even in low-risk populations. Maternal diet is also important for metabolic syndrome in this study, highlighting overall dietary patterns. Women with balanced, nutritious diets were at lower risk of metabolic abnormalities, a finding corroborated by Gao et al. (23), who found that higher diet quality was associated with a lower risk of gestational metabolic abnormalities. In the current analysis, maternal BMI was considered an environmental nutritional factor rather than a diagnostic component of metabolic syndrome in regression modelling, thereby reducing statistical overlap between the exposure and outcome variables. Pre-pregnancy BMI was a more reliable indicator of baseline metabolic risk, but data were incomplete and difficult to incorporate into the present analysis. On the same note, Pan et al. (24) also found that maternal glucose metabolism improves with better dietary quality during pregnancy. Zhang et al. (25) further supported these findings and demonstrated that dietary habits of early pregnancy are key determinants of the risk of gestational diabetes.

Further, Fan et al. (26) highlighted that maternal dieting affects both maternal and offspring metabolism, underscoring the long-term impacts of nutritional status. In our research, certain nutritional factors, such as dietary fibre and the glycemic index, were also linked to metabolic outcomes. Higher fibre consumption and low-glycemic diets were correlated with better metabolic profiles, which agrees with Sun et al. (27), who showed that dietary fibre supplementation can enhance glycemic control in pregnant women. Deng et al. (28) also affirmed that dietary interventions with a low glycemic index reduce the risk of adverse metabolic outcomes, thus emphasising the significance of dietary composition in controlling metabolic syndrome in pregnancy. Moreover, Petrella et al. (29) have found that the early dietary intervention in overweight and obese women is able to avoid the negative outcomes of pregnancy and, therefore, early nutritional treatment is required. Another significant determinant of metabolic syndrome identified in our study was physical activity.

Lower physical activity among women increased their likelihood of developing metabolic abnormalities, which conforms with Aburezq et al. (30), who found physical inactivity as a major risk of gestational metabolic disorders. Xie et al. (31) provided proof of a dose–response association between exercise and decreased chances of gestational diabetes, indicating that even small rises in exercise can be protective. Moreover, Schneider et al. (32) demonstrated that gestational diabetes risk could be reduced through physical activity’s effects on metabolic syndrome, thereby highlighting the interaction between lifestyle and metabolic health.

The association between maternal nutritional status and gestational weight gain observed in this study is supported by Ferreira et al. (33), who found that unhealthy dietary patterns are linked with excessive gestational weight gain. The weight gain is one of the factors that are known to contribute to metabolic syndrome and complications. Besides, Talebi et al. (34) found that an excessive amount of ultra-processed foods is linked to a high risk of adverse pregnancy outcomes, which supports the adverse effects of poor dietary habits. These results underscore the importance of dietary quality and caloric balance in maintaining optimal metabolic health during pregnancy. Our results also imply that maternal nutritional status is significant for offspring health, supporting the idea of fetal metabolic programming. Cohen et al. (35) showed that the quality of maternal diet is associated with the presence of hepatic fat in early childhood, which has long-term metabolic implications.

On the same note, Hill et al. (36) explained how maternal nutrition affects the development of the fetal pancreas, which can later predispose children to metabolic disorders. These observations highlight that enhancing maternal nutritional status may have long-lasting effects even after pregnancy. Besides the metabolic syndrome, our study results suggest a possible relationship between poor nutritional situation and hypertensive disorders of pregnancy. Imanpour et al. (37) also support this idea as they state that the risk of gestational hypertension and preeclampsia may be reduced by nutritional interventions.

Moreover, Asltoghiri et al. (38) demonstrated that metabolic markers in early pregnancy can predict adverse pregnancy outcomes, indicating that metabolic assessment at an early stage is essential for risk stratification. These results suggest that the underlying pathogenesis of metabolic syndrome and hypertensive disorders might have similarities associated with nutritional and metabolic imbalances. The general results of the research agree with the available evidence on larger scales, which associate diet and lifestyle with gestational metabolic disorders. Mijatovic-Vukas et al. (39) emphasised the interplay between dietary and physical activity in influencing the risk of gestational diabetes and the value of combined lifestyle measures.

Phalle and Gokhale (40) further asserted that dietary management is essential for improving maternal and fetal outcomes in gestational metabolic conditions. The combination of these findings points towards comprehensive approaches to maternal nutrition and lifestyle. In spite of these findings, there are some limitations which ought to be recognised. Nutritional data may be influenced by the recall bias, and the dietary intake assessment can be affected, as Pan et al. (24) also note. Also, although we find associations in our study, we cannot be sure of causal relationships. Future studies are recommended to use longitudinal designs and more detailed dietary assessment to gain insight into the temporal correlation between nutritional status and metabolic syndrome. Further investigation into micronutrient status and biochemical markers can also provide greater insight into the mechanisms underlying these associations.

The results should also be interpreted with caution for the possibility of reverse causality. Those pregnant women who were identified with metabolic abnormalities during antenatal care may have changed their dietary habits post-antenatal care after nutritional counselling or clinical recommendations. Thus, the dietary patterns observed in retrospective clinical data may, in part, reflect post-recognition dietary changes rather than dietary exposure at the beginning of the study.

### 4.1 Strengths of the study

The current study has several strengths that make the findings more valid and relevant. To begin with, it gives a global evaluation of maternal nutritional status with the integration of various dimensions, such as dietary patterns, physical activity, and metabolic indicators, and enables you to assess factors leading to metabolic syndrome during pregnancy in a holistic way. Secondly, the research addresses a significant and recent public health issue, namely the link between nutrition and metabolic syndrome, which is under-researched in most populations, especially low- and middle-income countries. Also, the inclusion of several variables enabled the detection of interrelated risk factors, providing a more comprehensive picture of how nutritional and lifestyle factors interact to produce metabolic effects. Evidence-based comparisons with the existing literature are also a strength of the study, enhancing the interpretability and external validity of the findings. In addition, the emphasis on early pregnancy determinants could provide clinical value, given the high likelihood of early detection and treatment of high-risk women.

### 4.2 Limitations of the study

Although this research has its advantages, it has some limitations that must be considered when reading the results. The first is that the study design does not provide opportunities to establish causal relationships between the mother’s nutritional status and the onset of metabolic syndrome, as the observed associations may be due to unmeasured confounding. Secondly, dietary intake was assessed mainly using a 24-h dietary recall and a food frequency questionnaire, both of which are susceptible to recall bias, reporting errors, and measurement error. Due to the retrospective abstraction of dietary data from clinical records, rather than under controlled research conditions, some degree of nutritional misclassification may have been present. Furthermore, pre-pregnancy BMI was not always recorded in the retrospective clinical data, and prenatal BMI was used as a surrogate marker of the mother’s nutritional status. The possibility that BMI during pregnancy could be affected by gestational weight gain cannot be fully ruled out. The associations were observed during routine antenatal care rather than using an established protocol to measure milestones over time, which could have contributed to the variability in timing of assessments. Because anthropometric measures during pregnancy may only partially reflect physiological GWG, some overlap between maternal adiposity and metabolic risk cannot be ruled out.

The study also lacked detailed biochemical assessment of micronutrient status, which could have provided additional insight into nutrient-specific metabolic effects. External factors such as socioeconomic status, cultural dietary practices, access to healthcare, and variations in physical activity may not have been fully controlled and could have influenced both nutritional status and metabolic outcomes. Physical activity assessment was relatively generalized and may not have adequately captured differences in intensity or duration among participants.

Although missing data were limited, retrospective record-based studies inherently carry the risk of incomplete documentation and the exclusion of records with insufficient data, which may introduce selection bias. Additionally, the study population may not fully represent broader populations, potentially limiting the generalizability of the findings. Despite these limitations, the biological plausibility of the observed associations is supported by consistency with previously published literature.

### 4.3 Future recommendations

Longitudinal study designs should be considered in future studies to more effectively determine causal relationships between maternal nutritional status and the development of metabolic syndrome during pregnancy. The validity of nutritional data could be enhanced by including objective, descriptive dietary assessment tools, such as food diaries or validated dietary indices. Also, in-depth biochemical analyses, such as micronutrient profiling and inflammatory markers, should be included in future research to gain deeper insight into the mechanisms underlying the interrelationships between nutrition and metabolic dysfunction. It is also necessary to investigate the efficacy of specific dietary interventions, including low-glycemic-index diets and increased dietary fibre intake, in preventing metabolic syndrome in high-risk pregnant women. Increasing the sample across different groups and contexts would enhance the external validity of the results and provide culturally sensitive data. Moreover, intervention-based research that analyses both diet and physical activity is advisable to assess their ability to reduce metabolic risks. Lastly, the combination of digital health solutions and tailored nutrition systems could provide new approaches to early diagnosis and treatment of metabolic syndrome during pregnancy.

## 5 Conclusion

In conclusion, the present study suggests a significant association between maternal nutritional status and the occurrence of metabolic syndrome in pregnancy. Dietary inadequacy, insufficient intake of nutrients, and less exercise were found to be significant factors leading to metabolic dysregulation in pregnant women. These findings suggest that maternal nutrition and lifestyle factors may represent important considerations in antenatal metabolic risk assessment and preventive health strategies. Early identification of women at increased metabolic risk may support targeted nutritional counselling and lifestyle-focused antenatal interventions, although prospective studies are needed to confirm causality and the effectiveness of interventions. On the whole, this paper shows that a holistic and proactive strategy towards maternal health, with nutrition being a primary element in ensuring healthy pregnancies and lifelong metabolic health, is necessary. Further prospective and longitudinal studies using standardized nutritional assessment methods are required to validate these findings and clarify temporal relationships between maternal nutrition and metabolic syndrome during pregnancy.

## Data Availability

The raw data supporting the conclusions of this article will be made available by the authors, without undue reservation.

## References

[1] Grieger JA Bianco-Miotto T Grzeskowiak LE Leemaqz SY Poston L McCowan LM . Metabolic syndrome in pregnancy and risk for adverse pregnancy outcomes: a prospective cohort of nulliparous women. PLoS Med. (2018) 15:e1002710. doi: 10.1371/journal.pmed.1002710, 30513077 PMC6279018

[2] Corrales P Vidal-Puig A Medina-Gomez G. Obesity and pregnancy, the perfect metabolic storm. Eur J Clin Nutr. (2021) 75:1723–34. doi: 10.1038/s41430-021-00914-5, 33911209

[3] Aldridge E Pathirana M Wittwer M Sierp S Leemaqz SY Roberts CT . Prevalence of metabolic syndrome in women after maternal complications of pregnancy: an observational cohort analysis. Front Cardiovasc Med. (2022) 9:853851. doi: 10.3389/fcvm.2022.853851, 35360031 PMC8963931

[4] Khammarnia M Ansari-Moghaddam A Kakhki FG Clark CCT Barahouei FB. Maternal macronutrient and energy intake during pregnancy: a systematic review and meta-analysis. BMC Public Health. (2024) 24:478. doi: 10.1186/s12889-024-17862-x, 38360655 PMC10870573

[5] Melero V Arnoriaga M Barabash A Valerio J Del Valle L Martin O’Connor R . An early mediterranean-based nutritional intervention during pregnancy reduces metabolic syndrome and glucose dysregulation rates at 3 years postpartum. Nutrients. (2023) 15:3252. doi: 10.3390/nu15143252, 37513670 PMC10383706

[6] Shen H Chen D Wang S Jin Y Cheng W. Effects of dietary fiber on maternal health in pregnant women with metabolic syndrome risk: a randomized controlled trial. Food Funct. (2024) 15:6597–609. doi: 10.1039/D3FO05120J, 38809131

[7] Bodnar LM Jin Q Naimi AI Simhan HN Catov JM Parisi SM . Periconceptional dietary quality and metabolic syndrome at 3 years postpartum. J Am Heart Assoc. (2024) 13:e035555. doi: 10.1161/JAHA.124.035555, 39158564 PMC11963925

[8] Tareke AA Melak EG Mengistu BK Hussen J Molla A. Association between maternal dietary diversity during pregnancy and birth outcomes: evidence from a systematic review and meta-analysis. BMC Nutr. (2024) 10:151. doi: 10.1186/s40795-024-00960-9, 39543687 PMC11566373

[9] Shi Y Zhong H Pang L. Maternal micronutrient disturbance as risks of offspring metabolic syndrome. J Trace Elem Med Biol. (2023) 75:127097. doi: 10.1016/j.jtemb.2022.127097, 36272194

[10] González-Fernández D Muralidharan O Neves PA Bhutta ZA. Associations of maternal nutritional status and supplementation with fetal, newborn, and infant outcomes in low-income and middle-income settings: an overview of reviews. Nutrients. (2024) 16:3725. doi: 10.3390/nu16213725, 39519557 PMC11547697

[11] Hasbullah FY Mohd Yusof BN Abdul Ghani R Mat Daud Z Appannah G Abas F . Maternal and dietary factors are associated with metabolic syndrome in women with a previous history of gestational diabetes mellitus. Int J Environ Res Public Health. (2022) 19:16797. doi: 10.3390/ijerph192416797, 36554678 PMC9779785

[12] Bartáková V Chalásová K Pácal L Ťápalová V Máchal J Janků P . Metabolic syndrome prevalence in women with gestational diabetes mellitus in the second trimester of gravidity. J Clin Med. (2024) 13:1260. doi: 10.3390/jcm13051260, 38592122 PMC10932344

[13] Michelsen TM Skytte HN Gunnes N Holven KB Christensen JJ Roland MCP. Metabolic profiles in early pregnancy associated with metabolic pregnancy complications in women with obesity. J Reprod Immunol. (2024) 166:104397. doi: 10.1016/j.jri.2024.104397, 39577057

[14] Kwok J Speyer LG Soursou G Murray AL Fanti KA Auyeung B. Maternal metabolic syndrome in pregnancy and child development at age 5: exploring mediating mechanisms using cord blood markers. BMC Med. (2023) 21:124. doi: 10.1186/s12916-023-02835-5, 37013575 PMC10071709

[15] Wilkins E Wickramasinghe K Pullar J Demaio AR Roberts N Perez-Blanco KM . Maternal nutrition and its intergenerational links to non-communicable disease metabolic risk factors: a systematic review and narrative synthesis. J Health Popul Nutr. (2021) 40:20. doi: 10.1186/s41043-021-00241-2, 33902746 PMC8077952

[16] Chen LW Loy SL Tint MT Michael N Ong YY Toh JY . Maternal pregnancy diet quality, night eating, and offspring metabolic health: the GUSTO study. Pediatr Res. (2025) 97:1528–36. doi: 10.1038/s41390-024-03574-w, 39300274

[17] Rao S Kanade AN Joshi SR Yajnik CS. Community-specific modifications are essential for objective assessment of maternal dietary intake–Pune maternal nutrition study. Public Health Nutr. (2009) 12:1470–6. doi: 10.1017/S1368980008004424, 19105869

[18] Yaktine AL Rasmussen KM. Weight Gain During Pregnancy: Reexamining the Guidelines. Washington: National Academies Press (2010). 20669500

[19] Alberti KGM Zimmet P Shaw J. The metabolic syndrome—a new worldwide definition. Lancet. (2005) 366:1059–62. doi: 10.1016/S0140-6736(05)67402-8, 16182882

[20] Habibi N Mousa A Tay CT Khomami MB Patten RK Andraweera PH . Maternal metabolic factors and the association with gestational diabetes: a systematic review and meta-analysis. Diabetes Metab Res Rev. (2022) 38:e3532. doi: 10.1002/dmrr.3532, 35421281 PMC9540632

[21] Mohebi A Pathirana MM Khoja A Wittwer MR Lowe K Fisher D . Prevalence of metabolic syndrome among pregnant women: a systematic review and meta-analysis. Endocrine. (2025) 88:398–409. doi: 10.1007/s12020-025-04160-8, 39841354 PMC12069128

[22] Li S Ma S Yao X Liu P. Effects of metabolic syndrome on pregnancy outcomes in women without polycystic ovary syndrome. J Endocrine Soc. (2024) 8:bvae143. doi: 10.1210/jendso/bvae143, 39224458 PMC11368129

[23] Gao X Zheng Q Jiang X Chen X Liao Y Pan Y. The effect of diet quality on the risk of developing gestational diabetes mellitus: a systematic review and meta-analysis. Front Public Health. (2023) 10:1062304. doi: 10.3389/fpubh.2022.1062304, 36699870 PMC9868748

[24] Pan W Karatela S Lu Q Xie L Wu S Jing J . Association of diet quality during pregnancy with maternal glucose metabolism in Chinese women. Br J Nutr. (2023) 130:958–65. doi: 10.1017/S0007114523000107, 36744324

[25] Zhang S Ni X Qiao T Zhao D Shen L Liang Y. Pre-pregnancy and early pregnancy dietary patterns and gestational diabetes risk among Miao women in China. Front Nutr. (2025) 12:1663054. doi: 10.3389/fnut.2025.1663054, 41567328 PMC12815701

[26] Fan M Chu Y Zheng Y Zhang Z Hou M. Association of pregnancy diet with metabolic adverse outcomes in pregnant women and their children: a systematic review and meta-analysis. Ann Nutr Metab. (2025) 81:123–40. doi: 10.1159/000543423, 39837283 PMC12136529

[27] Sun J Wang J Ma W Miao M Sun G. Effects of additional dietary fiber supplements on pregnant women with gestational diabetes: a systematic review and meta-analysis of randomized controlled studies. Nutrients. (2022) 14:4626. doi: 10.3390/nu14214626, 36364883 PMC9658588

[28] Deng Y Yu J Tao A Liu J Wang Q Cao Y . Effect of low-glycemic index diet advice on pregnant outcomes in women with elevated risk of gestational diabetes mellitus: a meta-analysis of randomized controlled trails. Clin Nutr ESPEN. (2023) 57:501–9. doi: 10.1016/j.clnesp.2023.07.091, 37739699

[29] Petrella E Tamborrino V Di Cerbo L Neri I Facchinetti F. An early, customized low-glycemic-index diet prevents adverse pregnancy outcomes in overweight/obese women. Minerva Ginecol. (2018) 70:254–60. doi: 10.23736/S0026-4784.17.04156-9, 29083138

[30] Aburezq M AlAlban F Alabdulrazzaq M Badr H. Risk factors associated with gestational diabetes mellitus: the role of pregnancy-induced hypertension and physical inactivity. Preg Hypertens. (2020) 22:64–70. doi: 10.1016/j.preghy.2020.07.010, 32745722

[31] Xie W Zhang L Cheng J Wang Y Kang H Gao Y. Physical activity during pregnancy and the risk of gestational diabetes mellitus: a systematic review and dose-response meta-analysis. BMC Public Health. (2024) 24:594. doi: 10.1186/s12889-024-18131-7, 38395913 PMC10893683

[32] Schneider AK Leemaqz SY Dalton J Verburg PE Mol BW Dekker GA . The interaction between metabolic syndrome and physical activity, and risk for gestational diabetes mellitus. Acta Diabetol. (2021) 58:939–47. doi: 10.1007/s00592-021-01696-9, 33743081

[33] Ferreira LB Lobo CV Miranda AEDS Carvalho BDC Santos LCD. Dietary patterns during pregnancy and gestational weight gain: a systematic review. Padrões alimentares durante a gravidez e ganho de peso gestacional: Uma revisão sistemática. Rev Bras Ginecol Obstet. (2022) 44:540–7. doi: 10.1055/s-0042-1744290, 35483873 PMC9948295

[34] Talebi S Mehrabani S Ghoreishy SM Wong A Moghaddam A Feyli PR . The association between ultra-processed food and common pregnancy adverse outcomes: a dose-response systematic review and meta-analysis. BMC Preg Childbirth. (2024) 24:369. doi: 10.1186/s12884-024-06489-w, 38750456 PMC11097443

[35] Cohen CC Perng W Sauder KA Shapiro ALB Starling AP Friedman C . Maternal diet quality during pregnancy and offspring hepatic fat in early childhood: the healthy start study. J Nutr. (2023) 153:1122–32. doi: 10.1016/j.tjnut.2023.01.039, 36796482 PMC10196613

[36] Hill DJ Hill TG. Maternal diet during pregnancy and adaptive changes in the maternal and fetal pancreas have implications for future metabolic health. Front Endocrinol. (2024) 15:1456629. doi: 10.3389/fendo.2024.1456629, 39377073 PMC11456468

[37] Imanpour V Khoshhali M Goodarzi-Khoigani M Kelishadi R. Systematic review and meta-analysis of nutritional interventions to prevent of gestational hypertension or/and preeclampsia among healthy pregnant women. J Res Med Sci. (2023) 28:25. doi: 10.4103/jrms.jrms_89_22, 37213454 PMC10199370

[38] Asltoghiri M Moghaddam-Banaem L Behboudi-Gandevani S Rahimi Froushani A Ramezani Tehrani F. Prediction of adverse pregnancy outcomes by first-trimester components of metabolic syndrome: a prospective longitudinal study. Arch Gynecol Obstet. (2023) 307:1613–23. doi: 10.1007/s00404-023-06967-0, 36869203

[39] Mijatovic-Vukas J Capling L Cheng S Stamatakis E Louie J Cheung NW . Associations of diet and physical activity with risk for gestational diabetes mellitus: a systematic review and meta-analysis. Nutrients. (2018) 10:698. doi: 10.3390/nu10060698, 29849003 PMC6024719

[40] Phalle A Gokhale D. Maternal and fetal outcomes in gestational diabetes mellitus: a narrative review of dietary interventions. Front Glob Women's Health. (2025) 6:1510260. doi: 10.3389/fgwh.2025.1510260, 40078500 PMC11897047

